# Dehydrozingerone inhibits renal lipotoxicity in high‐fat diet–induced obese mice

**DOI:** 10.1111/jcmm.16828

**Published:** 2021-08-12

**Authors:** Eun Soo Lee, Jeong Suk Kang, Hong Min Kim, Su Jin Kim, Nami Kim, Jung Ok Lee, Hyeon Soo Kim, Eun Young Lee, Choon Hee Chung

**Affiliations:** ^1^ Department of Internal Medicine Yonsei University Wonju College of Medicine Wonju Korea; ^2^ Institution of Genetic Cohort Yonsei University Wonju College of Medicine Wonju Korea; ^3^ Department of Internal Medicine Soonchunhyang University Cheonan Hospital Cheonan Korea; ^4^ Institute of Tissue Regeneration College of Medicine Soonchunhyang University Cheonan Korea; ^5^ Astrogen Inc Daegu Korea; ^6^ Department of Anatomy Korea University College of Medicine Seoul Korea; ^7^ Western Seoul Center Korea Basic Science Institute Seoul Korea

**Keywords:** dehydrozingerone, diabetic nephropathy, inflammation, lipotoxicity, reactive oxygen species

## Abstract

Ectopic fat accumulation in the kidneys causes oxidative stress, inflammation and cell death. Dehydrozingerone (DHZ) is a curcumin analog that exhibits antitumour, antioxidant and antidiabetic effects. However, the efficacy of DHZ in diabetic nephropathy (DN) is unknown. Here, we verified the efficacy of DHZ on DN. We divided the experimental animals into three groups: regular diet, 60% high‐fat diet (HFD) and HFD with DHZ for 12 weeks. We analysed levels of renal triglycerides and urinary albumin and albumin‐creatinine ratio, renal morphological changes and molecular changes via real‐time polymerase chain reaction and immunoblotting. Furthermore, high glucose (HG)‐ or palmitate (PA)‐stimulated mouse mesangial cells or mouse podocytes were treated with DHZ for 24 h. As a result, DHZ markedly reduced renal glycerol accumulation and albuminuria excretion through improvement of thickened glomerular basement membrane, podocyte loss and slit diaphragm reduction. In the renal cortex in the HFD group, phospho‐AMPK and nephrin expression reduced, whereas arginase 2 and CD68 expression increased; however, these changes were recovered after DHZ administration. Increased reactive oxygen species (ROS) stimulated by HG or PA in podocytes was inhibited by DHZ treatment. Collectively, these findings indicate that DHZ ameliorates DN via inhibits of lipotoxicity‐induced inflammation and ROS formation.

## 1 INTRODUCTION

Obesity is a major global health issue.^1^ Obesity‐induced dyslipidaemia has characteristics including increased flux of free fatty acids, triglycerides (TGs), low‐density lipoprotein (LDL), apolipoprotein B levels and decreased high‐density lipoprotein‐cholesterol (HDL‐C).^2^, ^3^, ^4^ Moreover, it is an important modifiable vascular risk factor and contributes to diabetic vascular complications such as neuropathy, retinopathy and nephropathy in type 2 diabetes mellitus (T2DM).^5^

Diabetic nephropathy (DN) is a leading cause of end‐stage renal disease and occurs in approximately 30%–40% of patients with diabetes.^3^ A previous study based on animal models of diabetes demonstrated induced renal damage and exaggerated albuminuria in hypercholesterolaemia and obese mice.^6^, ^7^ In the kidney, renal mesangial cells and glomerular epithelial cells such as visceral podocytes possess triglyceride (TG)‐rich lipoprotein receptors. Excessively accumulated lipid induces macrophage infiltration, cellular injury and apoptosis via inflammatory pathway activation, ROS over production and increase in proinflammatory cytokines such as tumour necrosis factor (TNF)‐α, transforming growth factor (TGF)‐β and interleukin (IL)‐6.^8^, ^9^, ^10^, ^11^

Dehydrozingerone (DHZ) is structural analogue of curcumin (CUR) and isolated from *Zingiber officinale*.^12^ DHZ already known to has beneficial effects such as inhibition of tumour growth, ROS and lipid peroxidation.^13^, ^14^, ^15^, ^16^ Previously, we demonstrated the effect of DHZ in high‐fat diet (HFD)–induced obese mice. DHZ administration markedly reduced the body weight and lipid accumulation in adipose tissue and liver by increasing AMP‐activated protein kinase (AMPK) activity. Moreover, DHZ regulated fasting blood glucose and leptin levels in the HFD mice.^17^

However, despite these beneficial effects in the metabolic diseases, the DHZ efficacy and related mechanisms on DN are not clearly known. Therefore, we assessed the DHZ effects and mechanism on DN development in HFD‐induced obese mice.

## 2 MATERIALS AND METHODS

### 2.1 Reagents

1, 7‐Bis (4‐hydroxy‐3‐methoxyphenyl)‐1, 6‐heptadiene‐3, 5‐dione (DHZ) was purchased from Sigma‐Aldrich, and 1,7‐bis(4‐hydroxy‐3‐methoxyphenyl)‐1,6‐heptadiene‐3,5‐dione (CUR) was purchased from Santa Cruz Biotechnologies (Santa Cruz).

### 2.2 Animal experiments

The animal experiments were conducted by following the methods in a previous study.^17^ In brief, C57BL/6 mice (4‐week‐old male, 15 g) were purchased from Dae Han Bio Link Co. and were housed in cages placed in a room under 12‐h light/dark cycle and ambient temperature (22–24°C). Further, 8‐week‐old mice were randomly divided into the following three groups: Group I, regular diet fed (RD); Group II, HFD fed (HFD, 60% kcal from fat); and Group III, HFD with DHZ (100 mg/kg). Thereafter, their food intake and body weights were recorded every week. After 12 weeks, the animals were anaesthetized, and whole blood was collected via cardiac puncture. Whole blood was centrifuged at 377 *g* for 15 min in a refrigerated centrifuge for serum collection; the collected tissues were then separated for staining or molecular analysis and stored at −80°C for further analysis. This study was approved by the Institutional Animal Care and Use Committee of Yonsei University at the Wonju Campus (YWC‐121030‐1).

### 2.3 Urinary albumin and creatinine assessment

Twenty‐four‐hour urine samples were centrifuged at 377 *g* for 10 min. Urinary albumin and creatinine levels were analysed with the commercial Albuwell M and Creatinine Companion Kits in accordance with the manufacturer's instructions (Exocell Inc.). Albumin‐to‐creatinine ratio (ACR) was calculated by dividing albumin concentration by creatinine concentration.

### 2.4 Renal glycerol analysis

Triglycerides is an ester derived from glycerol and three fatty acids. The renal TG contents were determined from renal glycerol values. Briefly, ethanolic potassium hydroxide was added to the kidney tissues and incubated at 55°C for overnight. Then, added 50% ethanol was added to the tissue and centrifuged for 5 min. The supernatant was transferred to a new tube and 50% ethanol, and the mixture was vortexed. Later, the supernatant was transferred to a new tube, 1 M magnesium chloride was added to it, and then, the mixture was kept on ice for 10 min. The mixture was centrifuged for 5 min, and the supernatant was transferred to a new tube. To determine the glycerol content, reconstituted glycerol reagent (Sigma‐Aldrich) was added to the supernatant according to the manufacturer's instructions. The mixture was incubated for 15 min at room temperature and measured absorbance at 540 nm. Renal TG levels were calculated using the following formula:

$$TG\;\left(mg/g\;tissue\right)\;=\;cuvette\;triolein\;equivalent\;glycerol\;concentration\;\left(mg/dL\right)\;\times \;\left(10/30\right)\;\times \;\left(415/200\right)\;\times \;0.012\;\left(\mathrm{dL}\right)/tissue\;weight\;\left(g\right)$$

### 2.5 Renal non‐esterified fatty acid and cholesteryl ester analysis

Renal non‐esterified fatty acid (NEFA) (MyBioSource) and cholesteryl ester (Abcam) levels were calculated using a commercial ELISA kit. Briefly, reagent was added to dissected kidney tissue and homogenized at 4℃ for 2 h to extract the NEFA. Then, the sample was centrifuged at 15,928 *g* for 10 min at 4℃ and take the supernatant was used for detection. The working solution was added to the supernatant, and the optical density value was measured at 715 nm with microplate reader. For cholesteryl ester, a mixture of chloroform and isopropanol was added to the tissues and the resulting mixture was centrifuged at 15,928 *g* for 10 min at 4℃. The supernatant was transferred to a new tube and dried at 50℃. Assay buffer was added to dried sample and then was read at 570 nm.

### 2.6 Transmission electron microscopy

To evaluate slit pore numbers and glomerular basement membrane (GBM) thickness, we analysed renal ultra‐structures using a transmission electron microscope (JEM‐1200EX II, JEOL, Ltd.). In this study, 10–15 pictures were taken per mouse, and an experiment was conducted in a total of 6 mice per group. The samples were captured at 20,000× magnification.

### 2.7 Haematoxylin and eosin staining

Renal glomerular volume was estimated in haematoxylin and eosin (H&E)–stained kidney section. Each section was observed using an optical microscope equipped with a charge‐coupled device camera (PULNiX).

### 2.8 Quantitative real‐time PCR

Total RNA was extracted from the kidney, mesangial and podocyte cells using TRIzol reagent (Sigma‐Aldrich). cDNA (1.0 μg) was prepared from the total RNA using a commercially available kit (Toyobo). Next, quantitative real‐time PCR was performed using the SYBR Green PCR master mix (Applied Biosystems) on an ABI PRISM 7900HT sequence detection system (Applied Biosystems). The specific gene expressions were analysed using the following primers: mouse podocin: forward 5′‐AAG TGC GGG TGA TTG CTG CAG AAG‐3′, reverse 5′‐TGT GGA CAG CGA CTG AAG AGT GTG‐3′; mouse IL‐1β: forward 5′‐CAA CCA ACA AGT GAT ATT CTC CAT G‐3′, reverse 5′‐GAT CCA CAC TCT CCA GCT GCA‐3′; mouse sterol regulatory element‐binding protein‐1 (SREBP1): forward 5′‐GGA GCC ATG GAT TGC ACA TT‐3′, reverse 5′‐GCT TCC AGA GAG GAG GCC A‐3′; mouse fatty acid synthase (FAS): forward 5′‐AGA GAT CCC GAG ACG CTT CT‐3′, reverse 5′‐ GCC TGG TAG GCA TTC TGT AGT‐3′; mouse TGF‐β1: forward 5′‐CCT GTC CAA ACT AAG GC‐3′, reverse 5′‐GGT TTT CTC ATA GAT GGC G‐3′; mouse intercellular adhesion molecule (ICAM): forward 5′‐GGG ACC ACG GAG CCA ATT‐3′, reverse 5′‐CT CGG AGA CAT TAG AGA ACA‐3′; and mouse glyceraldehyde 3‐phosphate dehydrogenase: forward 5′‐TGA ACG GGA AGC TCA CTG‐3′, reverse 5′‐GCT TCAC CAC CTT CTT GAT G‐3′. ICAM and TGF‐β1 mRNA expressions were additionally confirmed to band using PCR.

### 2.9 Western blot analysis

Protein from the kidney tissue and cultured cells was analysed using PRO‐PREP protein extraction solution (iNtRON Biotechnology). The concentration of the extracted protein was measured using a Bicinchoninic Acid Protein Assay Kit (Pierce, Rockford). Western blotting was performed on 8%–12% SDS‐PAGE. The blots were incubated with the following primary antibodies: anti‐arginase 2, anti‐cluster of differentiation 68 (CD68), anti‐nuclear factor, erythroid 2 like 2 (Nrf2), anti‐haeme oxygenase‐1 (HO‐1), anti‐NADPH oxidase (NOX) 4, sterol regulatory element‐binding proteins (SREBP) 1, SREBP2, acetyl‐CoA carboxylase (ACC), Bcl2, Bcl‐2‐associated X protein (BAX) and anti‐β‐actin (all from Santa Cruz Biotechnology) as well as anti‐nephrin (Abcam), anti‐phospho (p)‐AMPK, and anti‐p‐p38 and anti‐p38 (Cell Signaling, Danvers). The blots were visualized using a chemiluminescence UVP BioSpectrum 600 imaging system and quantified with ImageJ program (National Institute of Mental Health, Bethesda).

### 2.10 Cell culture and treatment

Conditionally immortalized mouse podocytes were provided from Dr. Peter Mundel.^18^ Mouse podocytes were cultured at 33°C under permissive conditions in Dulbecco's modified Eagle's medium (DMEM) containing 10% foetal bovine serum (FBS) and 10 U/ml mouse recombinant interferon‐γ (Sigma‐Aldrich) to enhance the expression of the thermosensitive T antigen. Podocyte was differentiated under nonpermissive conditions at 37°C without interferon‐γ for 14 days. The cells were maintained under serum‐deprived conditions for 24 h, treated with 400 µM palmitate (PA) with or without 20 µM DHZ for 24 h and then harvested for the next assay.

Mouse mesangial (MES‐13) was purchased from American Type Culture Collection (ATCC, Manassas, VA). MES‐13 cells were cultured in DMEM containing 5.5 mM glucose, 1% antibiotics and 10% FBS. The cultured cells were then starved for 24 h and treated with HG (30 mM) or PA (250 μM) with or without DHZ (20 μM). To compare AMPK activation between CUR and DHZ, 20 μM concentration of each of the compounds was used for treatment.

### 2.11 Reactive oxygen species (ROS) measurement

Intracellular ROS generation was detected using 2′‐7′ dichlorofluorescein diacetate (CM‐H_2_DCF‐DA; Molecular Probes) fluorescent probe. Podocytes on a glass dish were loaded with 5 µM CM‐H_2_DCF‐DA for 20 min at 37°C. Excess dye was washed using PBS. The fluorescence intensity was measured using LV10i inverted confocal microscope, and the fluorescent image was assessed using ImageJ software (National Institute of Health, Bethesda).

### 2.12 Statistics

Statistical analysis was performed using Prism 5.0 (GraphPad Software). All data are presented as mean ± standard error of the mean (SEM). Student's *t* test was used for experiments with two groups. *p* < 0.05 indicated statistical significance.

## 3 RESULTS

### 3.1 DHZ reduces HFD‐induced renal damage

**FIGURE 1:**
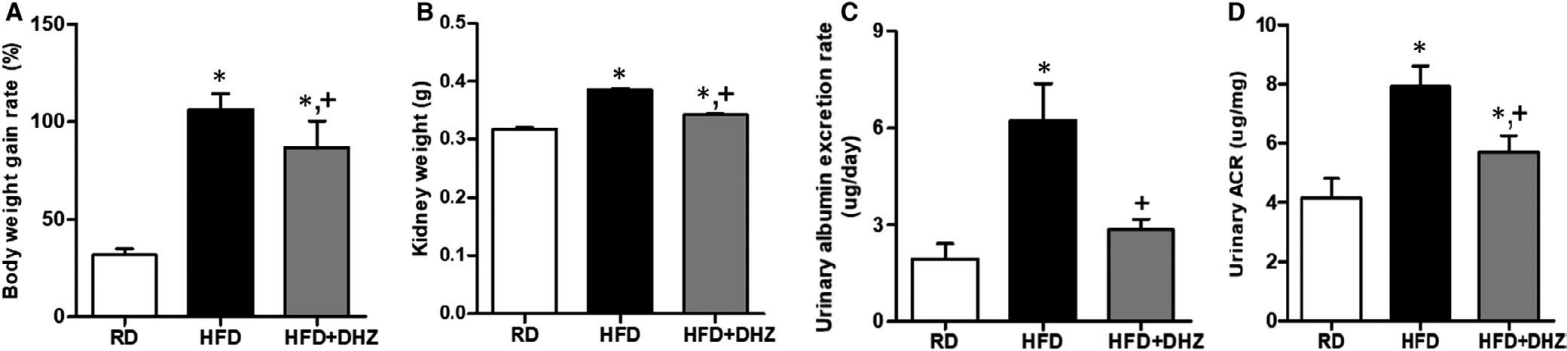
Analysis of body weight, kidney weight and urine collected at 24 h. The body weight gain rate (A) and kidney weight change (B) in each group. HFD and HFD with DHZ. The analysis of urinary albumin secretion rate and ACR (C) in urine collected at 24 h (D). ACR, albumin‐creatinine ratio; DHZ, dehydrozingerone; HFD, high‐fat diet; RD, regular diet. ^*^ *p* < 0.05 vs. RD, ^+^ *p* < 0.05 vs. HFD

**FIGURE 2:**
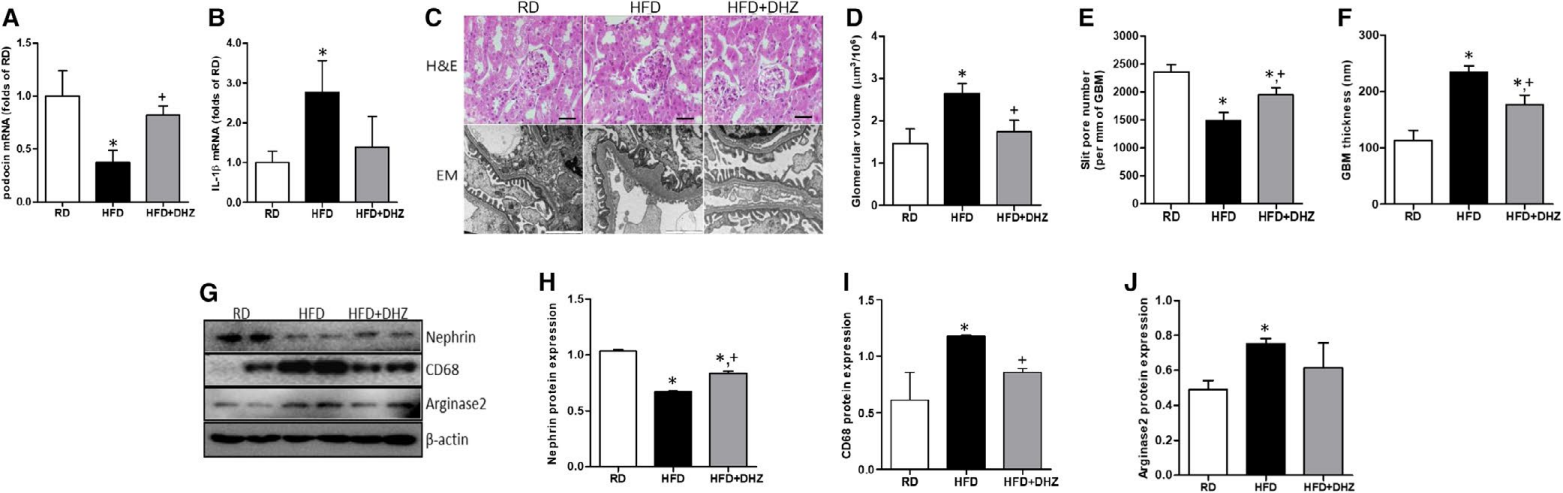
DHZ has renal protect in type 2 diabetic mice. The renal *podocin* and *IL*‐*1β* mRNA level changes were analysed via qRT‐PCR (A and B). Changes in glomerular volume and renal ultrastructure (C–F). Renal nephrin, CD68 and arginase 2 expression change were analysed via Western blotting (G–J). DHZ, dehydrozingerone; EM, electron microscopy; GBM, glomerular basement membrane; H&E, haematoxylin and eosin (magnification, 20 μm); HFD, high‐fat diet; RD, regular diet. ^*^ *p* < 0.05 vs. RD, ^+^ *p* < 0.05 vs. HFD

Both body and kidney weights of HFD‐administrated mice were increased (Figure 1A,B). The urinary albumin excretion for 24 h and ACR levels were significantly increased in HFD‐induced obese mice compared with RD mice. However, DHZ administration inhibited HFD‐induced albuminuria and increased (Figure 1C,D). Moreover, HFD administration reduced mRNA expression of *podocin* and increased the mRNA expression of the proinflammatory cytokine and *IL*‐*1β* (Figure 2A,B). It also disrupted the structures of glomeruli, GBM thickness and slit diaphragm (Figure 2C–F). Concomitantly, the expression of renal nephrin was decreased, while CD68 and arginase 2 expressions were increased; however, these changes were recovered in the DHZ‐administered HFD mice (Figure 2G–J). These data suggest that DHZ protects kidneys by inhibiting the disruption and inflammation of kidney in HFD‐induced obese mice.

### 3.2 DHZ improves dysregulated renal lipid metabolism

**FIGURE 3:**
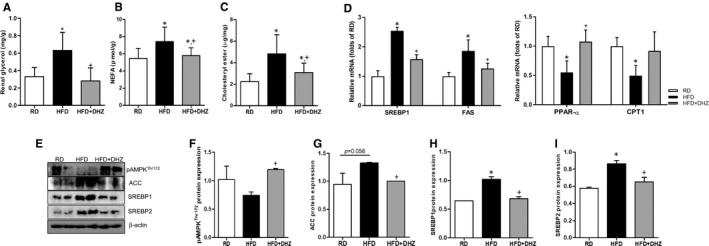
DHZ regulates lipid synthesis in kidney. Renal glycerol, NEFA and cholesteryl ester levels change by DHZ (A–C). Analysis of renal lipogenesis (*SREBP1 and FAS*) and lipolysis (*PPAR*‐*α and CPT1*)‐related gene mRNA levels via qRT‐PCR (D). pAMPK, ACC, SREBP1 and SREBP2 were regulated by DHZ administration (E–I). CPT1, carnitine palmitoyltransferase 1; DHZ, dehydrozingerone; FAS, fatty acid synthase; HFD, high‐fat diet; RD, regular diet; SREBP1, sterol regulatory element‐binding protein. ^*^ *p* < 0.05 vs. RD, ^+^ *p* < 0.05 vs. HFD

The DN has been associated with lipid accumulation in kidney. To evaluate inhibition of lipid accumulation by DHZ, we determined changes in the TG, NEFA and cholesteryl ester levels in the kidney of DHZ‐treated mice. Increased renal glycerol, NEFA and cholesteryl ester levels in HFD mice were markedly reduced following DHZ administration (Figure 3A–C). Moreover, increased *SREBP1* and *FAS* mRNA expression due to HFD administration was inhibited by DHZ administration. In contrast, DHZ administration mitigated decreased peroxisome proliferator‐activated receptor alpha *(PPAR*‐*α*) and carnitine palmitoyltransferase I (*CPT1*) levels in HFD mice (Figure 3D). In accordance with the previous studies demonstrating that the AMPK‐ACC pathway regulates fatty acid synthesis,^19^ HFD inhibited AMPK^Thr172^ phosphorylation and increased ACC expression in the kidney. Accordingly, expression levels of ACC, SREBP1 and SREBP2 were also increased; however, DHZ administration led to contradictory results such as increased AMPK phosphorylation and downregulated the expression levels of ACC, SREBP1 and SREPB2 (Figure 3E–I). PA‐induced lipid formation was reduced by DHZ treatment in both the kidney tissues and mesangial cells (Figure S1). These results indicate that DHZ regulates renal lipid metabolism in vivo.

### 3.3 DHZ inhibits inflammatory signals and ROS production in mouse mesangial cells

**FIGURE 4:**
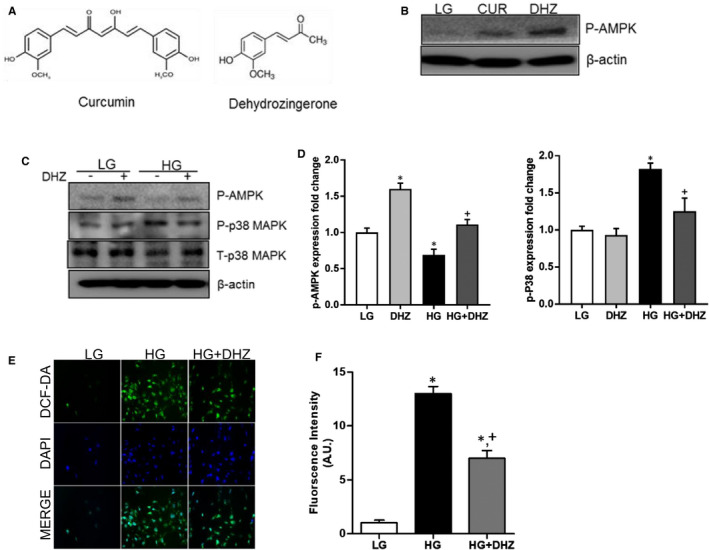
The effect of DHZ in mouse mesangial cells. CUR and DHZ chemical structures (A). Comparison of AMPK activity between CUR and DHZ (B). Regulation effects of pAMPK and p‐p38 MAPK expression by DHZ (C and D). ROS production changes by DHZ (E and F). CUR, curcumin; DHZ, dehydrozingerone; HG, high glucose; LG, low glucose; ROS, reactive oxygen species. ^*^ *p* < 0.05 vs. LG, ^+^ *p* < 0.05 vs. HG

A higher amount of AMPK was activated by DHZ than that by CUR at the same dosage (Figure 4A, B). HG‐induced reduction in pAMPK in mouse mesangial cells was recovered by DHZ treatment (Figure 4C). However, after AMPK inhibitor treatment with PA‐induced pAMPK reduction was not recovered despite of DHZ treatment (Figure S2A). Moreover, p38MAPK level induced by HG reduced following DHZ treatment (Figure 4D). Furthermore, PA‐induced pP38 MAPK‐pCREB‐COX2 levels also reduced by DHZ treatment (Figure 2B). DHZ treatment also reduced ROS levels following HG stimulation (Figure 4E,F). These data suggest that DHZ regulates AMPK and p38 MAPK signals and exerts anti‐inflammatory and antioxidant effects.

### 3.4 DHZ inhibits PA‐induced cellular toxicity via antioxidant effects in mouse podocytes

**FIGURE 5:**
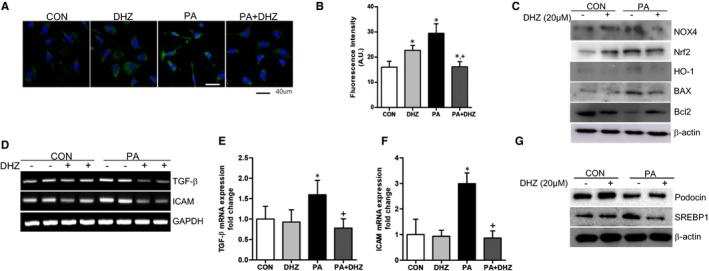
The effects of DHZ in PA‐treated mouse podocytes. DHZ effects against PA‐induced ROS formation in mouse podocytes (A). The NOX4, Nrf2/HO‐1 and Bcl/BAX protein expressions change by DHZ (B and C). DHZ reduction effect of PA‐induced *TGF*‐*β* and *ICAM* mRNA expression (D and E). PA‐induced podocin and SREBP1 expression changes recovered by DHZ (F and G). BAX, Bcl‐2‐associated X protein; DHZ, dehydrozingerone; HG, high glucose; HO‐1, haeme oxygenase‐1; ICAM, intercellular adhesion molecule 1; LG, low glucose; NOX, NADPH oxidase; Nrf2, nuclear factor erythroid 2‐related factor 2; TGF‐β, transforming growth factor‐beta. ^*^ *p* < 0.05 vs. LG, ^+^ *p* < 0.05 vs. PA

PA treatment increased ROS formation in mouse podocytes; however, PA with DHZ treatment inhibited PA‐induced ROS formation (Figure 5A, B). The expression of PA‐induced NOX4, antioxidant signal Nrf2/HO‐1 and BAX/Bcl2 was reduced by DHZ treatment (Figure 5C). Moreover, PA increased *TGF*‐*β* and *ICAM* mRNA expression also significantly reversed by DHZ treatment (Figure 5D–F). PA treatment reduced the expression of podocyte marker protein, podocin and upregulated SREBP1 expression. However, DHZ treatment inhibited these changes in mouse podocytes (Figure 5G). Collectively, these results indicate that DHZ exhibits protective effect against the PA‐induced cellular toxicity by regulating the oxidative stress and lipid metabolism.

## 4 DISCUSSION

The fundamental finding of this study is that DHZ inhibits DN incidence in a mouse model of HFD‐induced obesity via downregulating lipid accumulation and ROS production in the kidney. The increased prevalence of obesity is a major health challenge worldwide. Obesity induces insulin resistance, leading to abnormal lipid metabolism, inactivation of glucose transport in the muscle cells and elevated hepatic glucose production, thereby causing progression of type 2 diabetes.^20^, ^21^, ^22^

In DN, chronic hyperglycaemia plays a pivotal role via activation of several mechanisms, such as increasing TGF‐β expression, overproducing mesangial matrix and protein kinase C activation, which cause mesangial expansion via TGF‐β, vascular endothelial growth factor, ROS and angiotensin II. Moreover, abnormally increased glucose level induces lipid accumulation by activating DNMT1 CpG methylation and SREBP1c in the hepatocytes.^23^ Excessive ROS production caused by HG and abnormal lipid stimulation is associated with cell damage, eventually leading to cell death.^24^, ^25^

As a consequence of the aforementioned changes, lipids accumulate in the renal cells such as mesangial cells, podocytes and proximal tubule epithelial cells.^26^ These changes cause structural and functional deterioration of the kidney and lead to significant increase in urinary albumin excretion and mesangial expansion and decrease in glomerular filtration rate in patients with diabetes.^27^

Curcumin is a polyphenolic compound extracted from the rhizome of turmeric, and it is known to exert several beneficial effects.^28^ It possesses antioxidant, anti‐inflammatory and anticancer properties.^29^, ^30^ Moreover, it exerts antidiabetic effects via inducing glycaemic control and improves the hepatic antioxidant enzyme activities in mice with type 2 diabetes.^31^ Moreover, some studies have shown that CUR exerts beneficial effects in DN via regulating renal lipid metabolism and exhibiting anti‐inflammatory, antioxidant and anti‐fibrosis effects in animal model of T2DM.^32^, ^33^, ^34^

Curcumin is widely used as a dietary supplement; however, some studies have shown the potential adverse effects of CUR.^35^ Despite its significant oral intake, CUR is present in limited concentration in targets tissues and serum. Furthermore, it has poor bioavailability as it is not absorbed outside the gastrointestinal tract due to the presence of a β‐diketone moiety in its structure, which makes it instable and leads to its rapid metabolism.^36^ Moreover, some studies have reported that CUR may cause toxicity such as chromosome aberrations at concentration similar to those reported to exert beneficial effect.^37^, ^38^, ^39^

Dehydrozingerone is an analog of CUR without the β‐diketone moiety. Mapoung et al. showed that DHZ has superior effect in prostate cancer than CUR. Moreover, DHZ can exist in high concentration in the liver, kidneys and serum compared with CUR, suggesting that DHZ may not be metabolized rapidly in the liver after administration.^36^ In this study, we demonstrated that DHZ also has more beneficial effect in the kidney; however, future studies warranted to assess whether the aforementioned adverse effects are improved by DHZ.

Previously, we demonstrated the beneficial metabolic effects of DHZ in mouse models. DHZ inhibited liver and epididymal fat accumulation and regulated blood glucose via AMPK activation.^17^ In the present study, DHZ inhibited lipid synthesis in the kidney via reducing the expression of lipogenesis‐related genes including *SREBP1* and *FAS* and activated fatty acid oxidation‐related genes *PPAR*‐*α* and *CPT1*. Considering the other mechanisms involved in vascular complications, the incidence of DN correlated with increased levels of cellular glucose and lipid, and ROS formation by activated NADPH oxidase (NOX) that triggers inflammatory signals such as p38 MAPK, which play a pivotal role in cellular apoptosis.^40^, ^41^

In this study, PA stimulation increased ROS formation and activated NOX4 and BAX, which regulated cellular apoptosis. Co‐treatment with DHZ inhibited the expression of both NOX4 and BAX expression and activated antioxidant signalling and Nrf2/HO‐1 expression in the mouse podocytes. In mouse mesangial cells, AMPK phosphorylation was highly increased by DHZ than that by CUR. ROS generation and inflammatory response‐related p38 MAPK expression were activated by HG; however, these levels were reduced by DHZ treatment.

In DN, arginase 2 increases inflammatory macrophage infiltration to the kidney, which induces vascular dysfunction. Moreover, arginase 2 blockade protects DN by inhibiting renal macrophage infiltration AMP‐activated protein kinase.^42^ The present study demonstrated that DHZ administration recovered renal damage by inhibiting arginase 2 and CD68 expression and inflammatory cytokine mRNA levels in the kidney. However, DHZ did not affect the differentiation of bone marrow‐derived macrophages to M1 or M2 phenotype (Figure S3).

Although we confirmed that DHZ is more effective than CUR in in vitro experiments, we did not compare their effects in diabetic complications in the HFD mice. To develop therapeutics, we need to verify the efficacy of DHZ and CUR including their bioavailability and toxicity.

In conclusion, we demonstrated that HG‐ or PA‐induced ROS generation in mouse podocytes and mesangial cells is decreased by DHZ treatment. In podocytes, DHZ activated Nrf2/HO‐1 expression, whereas it reduced PA‐induced ROS formation. Moreover, DHZ protected the kidney against inflammatory signals and uncontrolled lipid metabolic signals both in vivo and in vitro. Due to these changes, increased albuminuria and disrupted kidney structures were markedly recovered in the HFD mice following DHZ treatment. Thus, collectively, our data suggest that DHZ is be a potential therapeutic agent for DN.

## CONFLICTS OF INTEREST

The authors declare no conflicts of interest.

## AUTHOR CONTRIBUTIONS

**EUNSOO LEE:** Conceptualization (lead); Data curation (lead); Funding acquisition (lead); Investigation (lead); Methodology (lead); Project administration (lead); Resources (lead); Software (lead); Validation (lead); Writing‐original draft (lead). **Jeong Suk Kang:** Data curation (equal); Formal analysis (supporting); Investigation (lead); Software (equal); Writing‐original draft (equal); Writing‐review & editing (equal). **Hong Min Kim:** Data curation (lead); Investigation (equal); Methodology (equal); Validation (supporting). **Su Jin Kim:** Formal analysis (supporting); Investigation (supporting); Methodology (equal). **Nami Kim:** Conceptualization (supporting); Investigation (supporting); Methodology (supporting). **Jung Ok Lee:** Investigation (equal); Methodology (equal); Supervision (supporting). **Hyeon Soo Kim:** Conceptualization (supporting); Investigation (supporting); Methodology (supporting); Project administration (equal); Validation (equal). **Eun Young Lee:** Data curation (supporting); Methodology (supporting); Supervision (supporting); Validation (equal); Writing‐review & editing (lead). **Choon Hee Chung:** Conceptualization (lead); Data curation (supporting); Funding acquisition (lead); Project administration (equal); Supervision (lead); Writing‐review & editing (lead).

## Supporting information

Supinfo S1

Supinfo S2

## Data Availability

The data supporting the findings of this study are available from the corresponding author upon reasonable request.

## References

[1] Flegal KM, Carroll MD, Ogden CL, Curtin LR. Prevalence and trends in obesity among US adults, 1999–2008. JAMA. 2010;303(3):235. 10.1001/jama.2009.2014 20071471

[2] Ginsberg HN, Huang LS. The insulin resistance syndrome: impact on lipoprotein metabolism and atherothrombosis. J Cardiovasc Risk. 2000;7(5):325‐331. 10.1177/204748730000700505 11143762

[3] Yuan CM, Nee R, Ceckowski KA, Knight KR, Abbott KC. Diabetic nephropathy as the cause of end‐stage kidney disease reported on the medical evidence form CMS2728 at a single center. Clin Kidney J. 2017;10(2):257‐262. 10.1093/ckj/sfw112 28396744 PMC5381235

[4] Athyros VG, Tziomalos K, Karagiannis A, Mikhailidis DP. Dyslipidaemia of obesity, metabolic syndrome and type 2 diabetes mellitus: the case for residual risk reduction after statin treatment. Open Cardiovasc Med J. 2011;5(1):24‐34. 10.2174/1874192401105010024 21660248 PMC3109607

[5] Haffner SM, American DA. Management of dyslipidemia in adults with diabetes. Diabetes Care. 2003;26(1):S83‐S86. 10.2337/diacare.26.2007.s83 12502625

[6] Utsunomiya K, Ohta H, Kurata H, Tajima N, Isogai Y. The effect of macrophage colony‐stimulating factor (M‐CSF) on the progression of lipid‐induced nephrotoxicity in diabetic nephropathy. J Diabetes Complications. 1995;9(4):292‐295. 10.1016/1056-8727(95)80025-A 8573750

[7] Mima A, Yasuzawa T, King GL, Ueshima S. Obesity‐associated glomerular inflammation increases albuminuria without renal histological changes. FEBS Open Bio. 2018;8(4):664‐670. 10.1002/2211-5463.12400 PMC5881532 29632818

[8] Wheeler DC, Fernando RL, Gillett MP, et al. Characterisation of the binding of low‐density lipoproteins to cultured rat mesangial cells. Nephrol Dial Transplant. 1991;6(10):701‐708. 10.1093/ndt/6.10.701 1754106

[9] Nishida Y, Oda H, Yorioka N. Effect of lipoproteins on mesangial cell proliferation. Kidney Int Suppl. 1999;71:S51‐S53. 10.1046/j.1523-1755.1999.07113.x 10412737

[10] Kramer A, Nauck M, Pavenstadt H, et al. Receptor‐mediated uptake of IDL and LDL from nephrotic patients by glomerular epithelial cells. Kidney Int. 1993;44(6):1341‐1351. 10.1038/ki.1993.387 8301935

[11] Wang L, Gill R, Pedersen TL, Higgins LJ, Newman JW, Rutledge JC. Triglyceride‐rich lipoprotein lipolysis releases neutral and oxidized FFAs that induce endothelial cell inflammation. J Lipid Res. 2009;50(2):204‐213. 10.1194/jlr.M700505-JLR200 18812596 PMC2636918

[12] Kubra IR, Bettadaiah BK, Murthy PS, Rao LJ. Structure‐function activity of dehydrozingerone and its derivatives as antioxidant and antimicrobial compounds. J Food Sci Technol. 2014;51(2):245‐255. 10.1007/s13197-011-0488-8 24493881 PMC3907648

[13] Desmarchelier C, del V. Pacciaroni A, Abate‐Daga D, Coussio J, Gil RR, Silva GL. Antioxidant and free radical scavenging activities ofMisodendrum punctulatum, myzodendrone and structurally related phenols. Phytother Res. 2005;19(12):1043‐1047. 10.1002/ptr.1786 16372370

[14] Rajakumar DV, Rao MN. Antioxidant properties of dehydrozingerone and curcumin in rat brain homogenates. Mol Cell Biochem. 1994;140(1):73‐79. 10.1007/BF00928368 7877600

[15] Parihar VK, Dhawan J, Kumar S, et al. Free radical scavenging and radioprotective activity of dehydrozingerone against whole body gamma irradiation in Swiss albino mice. Chem Biol Interact. 2007;170(1):49‐58. 10.1016/j.cbi.2007.07.006 17765885

[16] Rajakumar DV, Rao MN. Dehydrozingerone and isoeugenol as inhibitors of lipid peroxidation and as free radical scavengers. Biochem Pharmacol. 1993;46(11):2067‐2072. 10.1016/0006-2952(93)90649-H 8267655

[17] Kim SJ, Kim HM, Lee ES, et al. Dehydrozingerone exerts beneficial metabolic effects in high‐fat diet‐induced obese mice via AMPK activation in skeletal muscle. J Cell Mol Med. 2015;19(3):620‐629. 10.1111/jcmm.12455 25582026 PMC4369818

[18] Shankland SJ, Pippin JW, Reiser J, Mundel P. Podocytes in culture: past, present, and future. Kidney Int. 2007;72(1):26‐36. 10.1038/sj.ki.5002291 17457377

[19] O'Neill HM, Holloway GP, Steinberg GR. AMPK regulation of fatty acid metabolism and mitochondrial biogenesis: implications for obesity. Mol Cell Endocrinol. 2013;366(2):135‐151. 10.1016/j.mce.2012.06.019 22750049

[20] Martyn JA, Kaneki M, Yasuhara S. Obesity‐induced insulin resistance and hyperglycemia: etiologic factors and molecular mechanisms. Anesthesiology. 2008;109(1):137‐148. 10.1097/ALN.0b013e3181799d45 18580184 PMC3896971

[21] Wilcox G. Insulin and insulin resistance. Clin Biochem Rev. 2005;26(2):19‐39. 16278749 PMC1204764

[22] Shvartsval'd EP. Separate determination of the content of total bilirubin and bilirubin glucuronide in the capillary blood plasma. Lab Delo. 1973;11:649‐651. 4132562

[23] Wang Y, Chen L, Pandak WM, Heuman D, Hylemon PB, Ren S. High glucose induces lipid accumulation via 25‐hydroxycholesterol DNA‐CpG methylation. iScience. 2020;23(5):101102. 10.1016/j.isci.2020.101102 32408171 PMC7225732

[24] Kowluru RA, Mishra M, Kowluru A, Kumar B. Hyperlipidemia and the development of diabetic retinopathy: comparison between type 1 and type 2 animal models. Metabolism. 2016;65(10):1570‐1581. 10.1016/j.metabol.2016.07.012 27621192 PMC5023070

[25] Padilla A, Descorbeth M, Almeyda AL, Payne K, De Leon M. Hyperglycemia magnifies Schwann cell dysfunction and cell death triggered by PA‐induced lipotoxicity. Brain Res. 2011;1370:64‐79. 10.1016/j.brainres.2010.11.013 21108938 PMC3018544

[26] Gai Z, Wang T, Visentin M, Kullak‐Ublick GA, Fu X, Wang Z. Lipid accumulation and chronic kidney disease. Nutrients. 2019;11(4):722. 10.3390/nu11040722 PMC6520701 30925738

[27] Lim A. Diabetic nephropathy ‐ complications and treatment. Int J Nephrol Renovasc Dis. 2014;7:361‐381. 10.2147/IJNRD.S40172 25342915 PMC4206379

[28] Sahebkar A. Why it is necessary to translate curcumin into clinical practice for the prevention and treatment of metabolic syndrome? Biofactors. Mar‐Apr. 2013;39(2):197‐208. 10.1002/biof.1062 23239418

[29] Deck LM, Hunsaker LA, Vander Jagt TA, Whalen LJ, Royer RE, Vander Jagt DL. Activation of anti‐oxidant Nrf2 signaling by enone analogues of curcumin. Eur J Med Chem. 2018;143:854‐865. 10.1016/j.ejmech.2017.11.048 29223100

[30] Basnet P, Skalko‐Basnet N. Curcumin: an anti‐inflammatory molecule from a curry spice on the path to cancer treatment. Molecules. 2011;16(6):4567‐4598. 10.3390/molecules16064567 21642934 PMC6264403

[31] Seo KI, Choi MS, Jung UJ, et al. Effect of curcumin supplementation on blood glucose, plasma insulin, and glucose homeostasis related enzyme activities in diabetic db/db mice. Mol Nutr Food Res. 2008;52(9):995‐1004. 10.1002/mnfr.200700184 18398869

[32] Sun LN, Yang ZY, Lv SS, Liu XC, Guan GJ, Liu G. Curcumin prevents diabetic nephropathy against inflammatory response via reversing caveolin‐1 Tyr14 phosphorylation influenced TLR4 activation. Int Immunopharmacol. 2014;23(1):236‐246. 10.1016/j.intimp.2014.08.023 25196431

[33] Kim BH, Lee ES, Choi R, et al. Protective effects of curcumin on renal oxidative stress and lipid metabolism in a rat model of type 2 diabetic nephropathy. Yonsei Med J. 2016;57(3):664. 10.3349/ymj.2016.57.3.664 26996567 PMC4800357

[34] Ho C, Hsu YC, Lei CC, Mau SC, Shih YH, Lin CL. Curcumin rescues diabetic renal fibrosis by targeting superoxide‐mediated Wnt signaling pathways. Am J Med Sci. 2016;351(3):286‐295. 10.1016/j.amjms.2015.12.017 26992258

[35] Burgos‐Moron E, Calderon‐Montano JM, Salvador J, Robles A, Lopez‐Lazaro M. The dark side of curcumin. Int J Cancer. 2010;126(7):1771‐1775. 10.1002/ijc.24967 19830693

[36] Mapoung S, Suzuki S, Fuji S, et al. Dehydrozingerone, a curcumin analog, as a potential anti‐prostate cancer inhibitor in vitro and in vivo. Molecules. 2020;25(12):2737. 10.3390/molecules25122737 PMC7356390 32545675

[37] Nair J, Strand S, Frank N, et al. Apoptosis and age‐dependant induction of nuclear and mitochondrial etheno‐DNA adducts in Long‐Evans Cinnamon (LEC) rats: enhanced DNA damage by dietary curcumin upon copper accumulation. Carcinogenesis. 2005;26(7):1307‐1315. 10.1093/carcin/bgi073 15790590

[38] Cao J, Jia L, Zhou HM, Liu Y, Zhong LF. Mitochondrial and nuclear DNA damage induced by curcumin in human hepatoma G2 cells. Toxicol Sci. 2006;91(2):476‐483. 10.1093/toxsci/kfj153 16537656

[39] Urbina‐Cano P, Bobadilla‐Morales L, Ramirez‐Herrera MA, et al. DNA damage in mouse lymphocytes exposed to curcumin and copper. J Appl Genet. 2006;47(4):377‐382. 10.1007/BF03194648 17132903

[40] Kashihara N, Haruna Y, Kondeti VK, Kanwar YS. Oxidative stress in diabetic nephropathy. Curr Med Chem. 2010;17(34):4256‐4269. 10.2174/092986710793348581 20939814 PMC3708695

[41] Mittal M, Siddiqui MR, Tran K, Reddy SP, Malik AB. Reactive oxygen species in inflammation and tissue injury. Antioxid Redox Signal. 2014;20(7):1126‐1167. 10.1089/ars.2012.5149 23991888 PMC3929010

[42] Seok SJ, Lee ES, Kim GT, et al. Blockade of CCL2/CCR2 signalling ameliorates diabetic nephropathy in db/db mice. Nephrol Dial Transplant. 2013;28(7):1700‐1710. 10.1093/ndt/gfs555 23794669

