# Supplementary material for: Dehydrozingerone inhibits renal lipotoxicity in high‐fat diet–induced obese mice
Source: J Cell Mol Med. 2021 Aug 12;25(18):8725–33. doi: 10.1111/jcmm.16828 (PMC8435425; doi:10.1111/jcmm.16828)
Supplement: Supplementary file 1 — Supinfo S1 [file JCMM-25-8725-s002.doc]

**SUPPLEMENTARY MATERIALS AND METHODS**

**Reagents**

Compound C was purchased from Sigma-Aldrich (St. Louis, MO, USA) and Ro-318220 was obtained from Calbiochem (San Diego, CA, USA).

**Macrophage differentiation**

C57BL/6 strain 8weeks old age male mice were euthanized by cervical dislocation. And then peel the skin over the foot. Cut off the hind legs at the hip joint and place femurs in the PBS. Remove excess muscle from legs and carefully sever leg bones proximal to each joint. Attach 10-ml syringe to 25-G needle and fill with cold sterile PBS without calcium and magnesium. Insert needle into bone marrow cavity of femur and flush bone cavity with 2 to 5 ml of the PBS, until bone cavity appears white. Allow wash medium to collect in a sterile 50-ml conical centrifuge tube on ice. Then, centrifuge cells for 20 min at 1,000 rpm on room temperature. Separated supernatant was discard and suspend cell pellet in macrophage complete medium by pipetting up and down. Count total bone marrow progenitor cells in a hemacytometer and adjust cells to a concentration of 4 × 10^6^ /ml in macrophage complete medium (DMEM/F12 with 10% FBS and 100 U/ml recombinant M-CSF). Add a total of 2× 10^5^ cells in 6 well plates and incubate in 37°C, 5% CO_2_ incubator. After cell stabilizes, cells were stimulated with LPS (100 ng/ml), IFN-γ (25 ng/ml) for M1 differentiation, IL4 (20 ng/ml) for M2 differentiation with or without DHZ (20 μM). After 24 hrs, protein extracted from the cells and then CD68, CD206 and MCP1 levels were confirmed by western blotting.

**BODIPY staining**

Cultured mouse mesangial cells were stimulated with Palmitic acid (PA, 250 μM) for 24 hrs. At the time-point of interest, added 1 μM BODIPY staining solution to wells for 15min at 37°C. And then, remove media and wash cells using PBS to remove staining solution. Fix cells in 4% paraformaldehyde (PFA) for 30 min. Remove PFA and wash the wells 3 times for 5 min. Stained cells were mounted using fluoroshield mounting medium containing 4′,6-diamidino-2-phenylindole (Sigma, St Louis, Mo, USA). Fluorescence images were obtained using the laser scanning confocal microscope (LSM780, Zeiss, Oberkochen, Land Baden-Württemberg, Germany).

**Western blot analysis**

Protein from cultured mesangial cells were analyzed using PRO-PREP protein extraction solution (iNtRON Biotechnology, Korea). The concentration of the extracted protein was measured using a bicinchoninic acid protein assay kit (Pierce, Rockford, IL, USA). Western blotting was performed on 8–12% SDS-PAGE gel. The blots were incubated with the following primary antibodies: anti-phospho (p)-CREB, anti-CREB and COX2 (Cell signaling, Danvers, MA, USA). The blots were visualized using a chemiluminescence UVP BioSpectrum 600 imaging system and quantified with ImageJ program (National Institute of Mental Health, Bethesda, MD, USA).
