# Supplementary material for: Dehydrozingerone inhibits renal lipotoxicity in high‐fat diet–induced obese mice
Source: J Cell Mol Med. 2021 Aug 12;25(18):8725–33. doi: 10.1111/jcmm.16828 (PMC8435425; doi:10.1111/jcmm.16828)
Supplement: Supplementary file 2 — Supinfo S2 [file JCMM-25-8725-s001.doc]

**Supplementary information**

**Dehydrozingerone inhibits renal lipotoxicity in high-fat diet-induced obese mice**

Eun Soo Lee^1, 2#^, Jeong Suk Kang^3, 4#^, Hong Min Kim^5^, Su Jin Kim^6^, Nami Kim^7^, Jung Ok Lee^6^, Hyeon Soo Kim^6^, Eun Young Lee^3, 4*^ and Choon Hee Chung^1*^

^1^Department of Internal Medicine, Yonsei University Wonju College of Medicine, Wonju 26426, Gangwon-do, South Korea

^2^Institution of Genetic Cohort, Yonsei University Wonju College of Medicine, Wonju 26426, Gangwon-do, South Korea

^3^Department of Internal Medicine, Soonchunhyang University Cheonan Hospital, Cheonan 31151, Chungcheongnam-do, South Korea

^4^Institute of Tissue Regeneration, College of Medicine, Soonchunhyang University Cheonan Hospital, Cheonan 31151, Chungcheongnam-do, South Korea

^5^Astrogen Inc., Daegu 41072, Gyeongsangnam-do, South Korea

^6^Department of Anatomy, Korea University College of Medicine, Seoul 02841, Republic of Korea

^7^Western Seoul Center, Korea Basic Science Institute, Seoul 03759, Republic of Korea.

*To whom correspondence should be addressed : [cchung@yonsei.ac.kr](mailto:cchung@yonsei.ac.kr) or Eun Young Lee, Email: eylee@sch.ac.kr


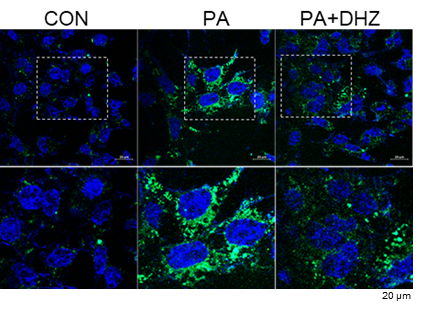


**Supplementary Figure 1.** Lipid accumulation inhibits effect of DHZ in MES-13.

BODIPY staining in MES-13. PA, palmitate; DHZ, dehydrozingerone (scale bar=20 μM).


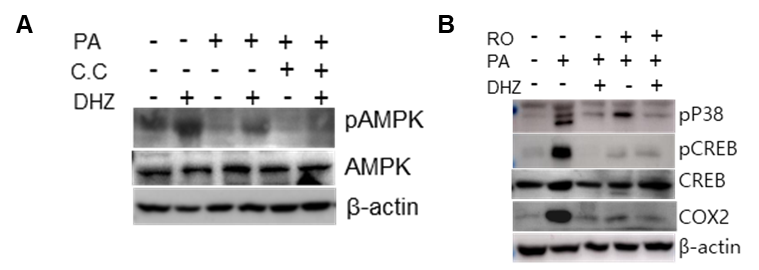


**Supplementary Figure 2.** Regulation of AMPK and p38 MAPK signals by DHZ.

Western blot analysis of pAMPK, AMPK, pP38, pCREB and COX2 expression. PA, palmitate; C.C, Compound C; RO, RO318220; DHZ, dehydrozingerone.


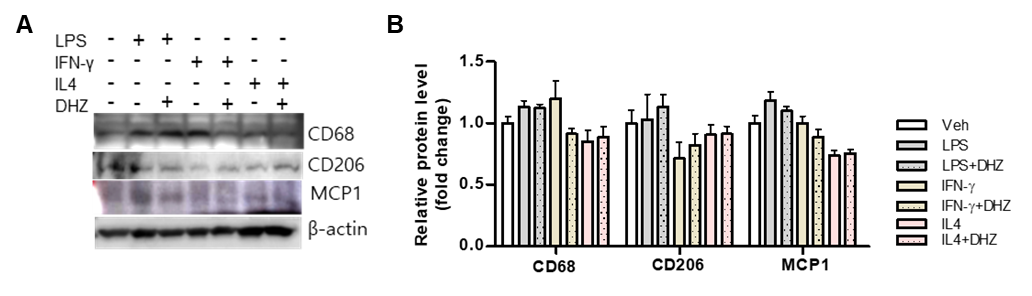
**Supplementary Figure 3.** The DHZ effects of macrophage differentiation in bone marrow derived macrophage.

CD68, CD206 and MCP1 expression change by LPS and IFN-γ stimulation for M1 differentiation and IL4 simulation for M2 differentiation with or without DHZ. LPS, Lipopolysaccharides; IFN-γ, interferone-γ; IL-4, interleukin-4; DHZ, dehydrozingerone.
